# Geranylgeranylacetone as Prevention for Postoperative Atrial Fibrillation (GENIALITY)

**DOI:** 10.1007/s10557-025-07693-2

**Published:** 2025-04-14

**Authors:** Kennedy S. Ramos, Soufiane Nassiri, Leonoor F. J. Wijdeveld, Reinier L. van der Palen, Myrthe F. Kuipers, Mellanie True Hills, Pieter Slijkerman, Daniel H. van Raalte, M. Louis Handoko, Natasja M. S. de Groot, Nimrat Grewal, Robert J. M. Klautz, Etto C. Eringa, Bianca J. J. M. Brundel

**Affiliations:** 1 Amsterdam Cardiovascular Sciences, Heart Failure and Arrhythmias, Amsterdam University Medical Center, Location Vrije Universiteit Amsterdam, De Boelelaan 1117, Physiology, 1081 HV Amsterdam The Netherlands; 2 Department of Cardiology, Amsterdam University Medical Center, Amsterdam, The Netherlands; 3 AFIP Foundation, Amsterdam, The Netherlands; 4 Stopafib.Org, American Foundation for Women’S Health, Decatur, TX USA; 5 Innovation Exchange Amsterdam, Amsterdam University Medical Center, Amsterdam, The Netherlands; 6 Diabetes Center, Department of Internal Medicine, Amsterdam University Medical Center, Amsterdam, The Netherlands; 7 Department of Cardiology, Erasmus Medical Center, Rotterdam, The Netherlands; 8 Department of Cardiothoracic Surgery, Amsterdam University Medical Center, Amsterdam, The Netherlands; 9 Department of Cardiothoracic Surgery, Leiden University Medical Center, Leiden, The Netherlands; 10 Department of Physiology, Cardiovascular Research Institute Maastricht, Maastricht, The Netherlands

**Keywords:** Heat shock proteins, Prevention, Cardiothoracic surgery, Postoperative atrial fibrillation

## Abstract

**Abstract:**

**Purpose:**

Interestingly, 30–50% of patients undergoing elective cardiothoracic surgery develop postoperative AF (PoAF). Unfortunately, preventive PoAF therapy is still suboptimal. In our previous studies, we showed that oral Geranylgeranylacetone (GGA) administration increased cardioprotective heat shock protein (HSP) protecting against AF onset and progression in clinically relevant animal model studies.

**Methods:**

The GENIALITY study is a phase II single-center, double-blind, placebo-controlled randomized trial comparing the efficacy of GGA in preventing PoAF. Participants (*N* = 146) are adult patients, without any registered history of AF, undergoing elective open-heart surgery for valvular disease, coronary artery bypass grafting, or concomitant, and are allocated with ratio 1:1 in treatment or placebo groups. Daily administration of 300 mg of GGA or placebo starts 5 days before until 3 days after surgery. Cardiac rhythm will be monitored using a Holter monitoring post-surgery until hospital discharge. Additionally, blood samples, right atrial appendage tissue, and epicardial adipose tissue will be collected to assess proteostasis levels.

**Results:**

The primary endpoint is the assessment of PoAF incidence in the GGA group compared to the placebo group. Secondary endpoints include the evaluation of HSP levels through biochemical analysis in both blood and atrial tissue.

**Conclusion:**

The GENIALITY study aims to reduce PoAF incidence in the GGA group compared to the placebo group. Herewith, we expect to obtain proof of concept for a beneficial effect of GGA in preventing PoAF in patients undergoing cardiothoracic surgery.

**Trial Registration:**

Clinical Trial Information System (CTIS) registry: 2024–514743-28–00. Authorized on September 30th 2024.

## Introduction

Atrial fibrillation (AF) is the most common cardiac arrhythmia in Western countries, with a predicted prevalence of nearly 18 million diagnosed patients in 2060 [1]. AF is a progressive disease associated with an increased risk of stroke, heart failure, and mortality [2]. AF progression is rooted in the combination of structural and electrical atrial remodeling [3].

Postoperative AF (PoAF) is defined as new-onset AF in the immediate period following cardiothoracic surgery and is the most common complication following cardiac surgeries [4, 5]. The incidence of PoAF ranges between 30 and 50% in patients undergoing cardiac surgical procedures [4, 6]. Common characteristics involve an onset occurring 2–4 days after surgery [4], with episodes that are typically brief and self-terminating. Associated clinical and epidemiological consequences of PoAF include increased risk of stroke, hemodynamic instability, lengthened hospital and intensive care unit stays, and higher (or increased) health-care costs [1]. Although the European Society of Cardiology recommends prophylactic use of ß-blockers for patients at risk of PoAF [1], there is still a lack of consistent data from large randomized clinical trials supporting the efficacy of such medications in reducing the incidence of PoAF. This underscores the urgent need to identify effective drugs for PoAF prevention. Moreover, reason for suboptimal treatment is that the underlying mechanisms for PoAF are incompletely understood. Potential pathophysiological mechanisms involved include inflammation, sympathetic activation, and cardiac ischemia. These factors collectively may trigger PoAF, especially in parallel to pre-existing factors, including atrial electropathology that render the atria susceptible to AF onset and progression [7].

Emerging research findings reveal a key role for protein homeostasis (proteostasis) derailment to drive AF. A balanced proteostasis is of vital importance for the proper function of cardiomyocytes and the heart overall [3, 8]. Given the limited regenerative capacity in long-lived postmitotic cardiomyocytes, proteostasis plays a role of paramount significance to maintain a healthy heart function. During stress, including cardiac surgery, cardiomyocytes respond by inducing a heat shock response (HSR), by activating heat shock protein (HSP) expression. HSPs are chaperones capable of binding to proteins such as cytoskeletal and sarcomere protein structures and thereby preventing them from misfolding and disruption and as such protect against electropathology and AF onset and progression [9–13]. Previous research findings disclosed that especially small HSPs bind to myofibrils and protect against myofibril degradation in experimental and clinical AF [9, 11, 14]. Clinical studies show that the higher expression of HSPB1 in atrial tissue samples was associated with short duration of AF and with minimal degradation of sarcomere protein structures [9, 10, 15]. In line, research showed that the derailment of the HSR and exhaustion of atrial HSP levels play an important role underlying electropathology and AF progression [9, 10, 16].

Hence these molecular mechanistic insights pave importance to HSR boosting as a novel pharmacological strategy to reduce AF risk. The HSP-inducing compound geranylgeranylacetone (GGA) may present as a suitable candidate. GGA likely confers its cardioprotective effects on AF, by enhancing HSF1 hyperphosphorylation with the subsequent induction of cardioprotective HSP expression [15]. The cardioprotective effect of GGA is likely via the increased expression of HSPB1, as ablation of this HSP diminishes the protective effect of GGA [10, 15]. HSPB1 binds to structural proteins and as such conserves the cardiomyocyte structure and function [8, 10, 14]. In addition, GGA enhances protein and functional recovery in an in vitro model for AF [11]. The small signaling protein RhoA appears to be engaged in this mechanism, mediated by C20 (geranylgeranyl) isoprenoids via its post-translational prenylation [17]. GGA may compete with endogenous geranyl groups affecting RhoA activation and consequently enhancing HSP expression. Interestingly, previous research raised importance of the geranyl group within the GGA structure, given that a loss of HSP induction was observed in the administration of GGA derivates with farnesyl isoprenoid (C15) [15].

Additionally, GGA has been shown to enhance cardiomyocyte HSP expression levels. In an in vitro AF atrial cardiomyocyte model system, the administration of GGA boosted HSP expression and protected against contractile dysfunction [14]. In a *Drosophila* AF model, GGA protected against myocardial dysfunction and increased arrhythmicity [14]. The administration of GGA in dog models for rapid-pacing and acute ischemia-induced AF protected the heart against electrophysiological impairment, contractile dysfunction, and AF onset and progression [9, 18]. Furthermore, protective effects of HSPs against AF inducibility were observed in a rabbit heart failure model system [19]. Importantly, a placebo-controlled clinical trial in patients with coronary artery disease, administering orally 400 mg/day of GGA for 3 days prior to coronary artery bypass grafting surgery, already resulted in a significant increase in HSP expression in the right and left atrial appendages compared to placebo-treated patients. Moreover, increased HSP levels, especially HSPB1, were present at the myofilaments in patients treated with GGA, suggesting a beneficial effect by the conservation of the myofilament structures [14].

Based on the observed cardioprotective findings of GGA, in the current phase II trial, we aim to test a beneficial effect of GGA in preventing PoAF in patients undergoing cardiothoracic surgery.

## Study Design

**Fig. 1:**
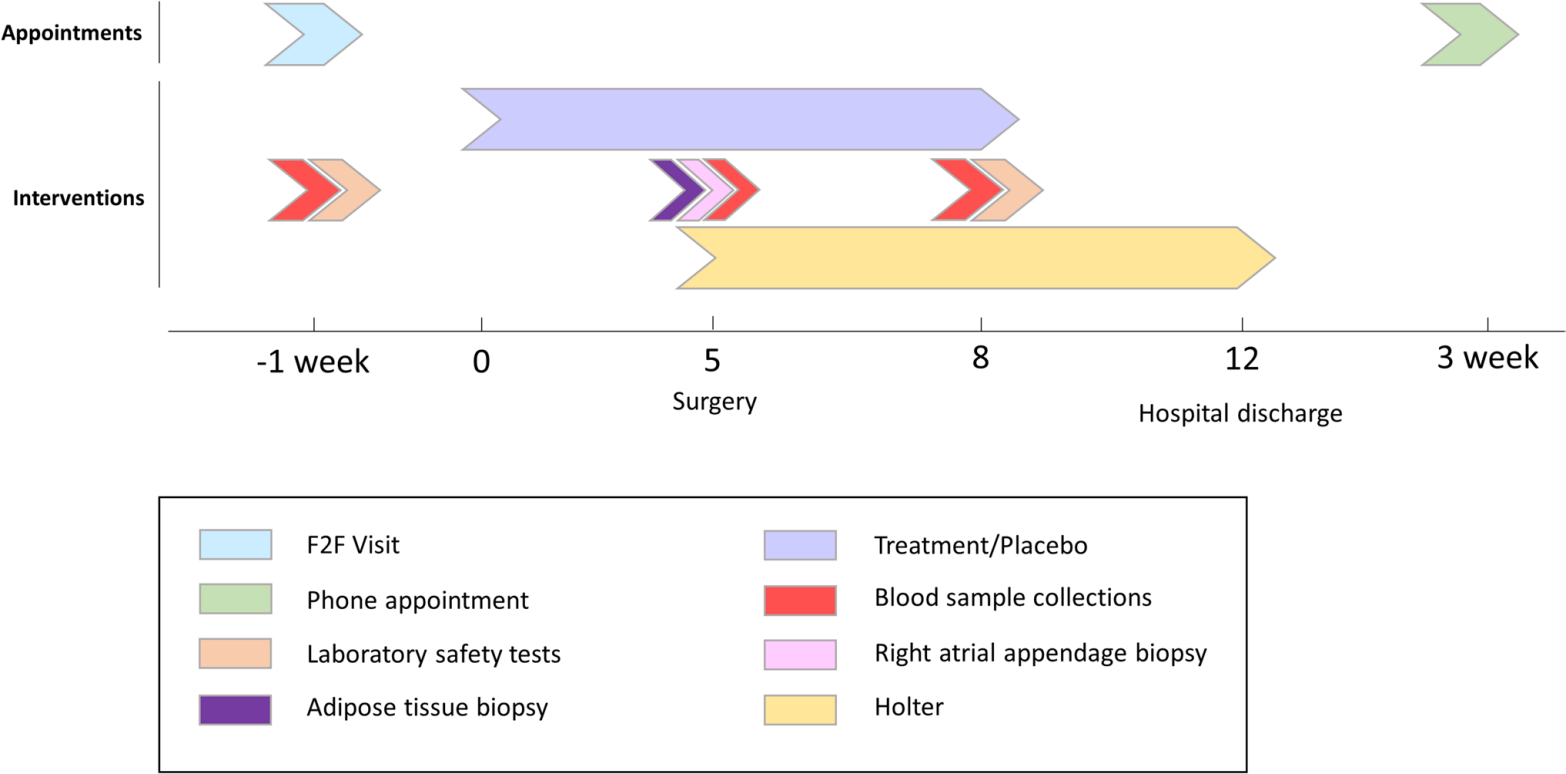
Study timeline

The GENIALITY study is a prospective interventional trial, with a scheduled duration of 18 months. After eligibility is checked and informed consent (IC) is obtained, patients are invited for a baseline visit 1 week before scheduled cardiothoracic surgery. During this visit, a physical examination and blood collection are performed, and instructions about treatment/placebo administration are provided. Five days before and until 3 days after cardiothoracic surgery, participants are instructed to start 2 mL/day of treatment administration, either placebo (2 mL Miglyol 812 N) or GGA solution (300 mg GGA in 2 mL of Miglyol 812 N). Although the standard “on-label” dosage for the treatment of stomach ulcers and gastritis is 150 mg/day [20], a higher dosage of 300 mg/day was safe for participants in other clinical trials [21, 22] and is closer to the dose of 400 mg/day for 3 days chosen by van Marion et al. [14]. Immediately before the commencement of cardiac surgery, a new blood sample is collected, which is processed into serum and stored at − 80 °C. Atrial tissue samples, collected from the right atrial appendage at the cannulation incision for extracorporeal circulation, and epicardial adipose tissue are immediately snap-frozen in liquid nitrogen and stored at − 80 °C. Immediately after surgery, the patient’s heart rhythm is continuously monitored by the Holter device until hospital discharge—usually on the third day post-surgery—or until the end of the 7th day post-surgery. Holter data are collected for further analysis by research staff to identify the incidence of PoAF longer than 10 s. While previous consensus statements defined sustained AF as lasting longer than 30 s [1], recent discussions emphasize the importance of reconsidering shorter thresholds in the context of continuous rhythm monitoring technology available today [23, 24]. After the last day of treatment/placebo administration, the third blood sample is collected. Three weeks after surgery, patients are contacted by phone for a follow-up appointment. Per patient, the protocol is depicted in Fig. 1. At the time of publication, the study is in the active enrollment phase.

The study is carried out in adherence to the Declaration of Helsinki and in accordance with the Medical Research involving Human Subjects Act. Also, approval was granted by the local medical ethical committee of the Amsterdam University Medical Centra with the number MEC 2024.1014, recorded in the Clinical Trial Information System (CTIS) registry with the trial number 2024–514743-28-00.

### Study Objectives

The primary objectives are to evaluate whether GGA administration reduces the incidence of PoAF in patients undergoing open-chest cardiac surgery compared to a placebo. The secondary objective is to investigate the activation of the HSR by measuring HSP levels after GGA treatment compared to a placebo in blood and atrial adipose and myocardial tissue samples.

### Study Population

The study population (*N* = 146) consists of adults (18–80 years old) who never had documented history of AF. Patients who are diagnosed with mitral valve stenosis or regurgitation, and/or aortic valve stenosis or regurgitation, and/or coronary artery disease are scheduled for valve repair/replacement and/or coronary artery bypass grafting (CABG) at Amsterdam UMC.

### Inclusion Criteria

In order to be eligible to participate in this study, a subject must meet all of the following criteria:

- Aged between 18 and 80 years old.
- Elected for cardiothoracic surgery for mitral valve stenosis or regurgitation, and/or aortic valve stenosis or regurgitation, and/or coronary artery disease

### Exclusion Criteria

A potential participant who meets any of the following criteria is excluded from participation in this study:

- Documented history of AF
- Not able to undergo the complete study protocol
- Previous cardiothoracic surgery
- Doubt about compliance
- Pre-menopausal women who are nursing or pregnant
- Chronic malabsorption
- Any documented or suspected malignancy or history of malignancy within 1 year prior to screening, except appropriate treated basal cell carcinoma or in situ carcinoma of the cervix
- Currently enrolled in another drug trial

### Sample Size Calculation

Based on our study population, the expected incidence of PoAF in our study population is 32% [25]. The sample size calculation of the present study design was based on dichotomous outcomes for two independent groups (treatment and placebo), as this parameter has an expected incidence drop from 32 to 12%, where alpha = 5%, beta = 0.2, and power = 80% and 2-sided testing [R Statistics 3.6.3, pwr-package 1.3–0]. Therefore, the initial required number of patients undergoing cardiac surgery is 66 in the GGA-treated group and 66 in the placebo group. An attrition rate of 10% increases this to 73 patients in the GGA-treated group and 73 in the placebo group.

### Continuous Heart Monitoring (Holter)

Immediately after cardiothoracic surgery, the heart rhythm of each patient is continuously monitored by a Holter until hospital discharge. In the case of post-operative hospitalization lasting longer than 7 days, Holter examination is discontinued by the end of the 7th day.

### Blood Sampling

During the baseline appointment, immediately before surgery, and on day 8 after the start of treatment/placebo, blood samples are collected and processed into serum, ultimately stored at − 80 °C.

### Atrial Tissue Sampling

During cardiothoracic surgery, after the introduction of the extra corporal circulation into the right atrium via the right atrial appendage (RAA), an atrial tissue sample (approximately 10 × 10 mm) is obtained from the incision site in all patients. Collected RAA samples are immediately snap-frozen in liquid nitrogen and stored at − 80 °C.

### Epicardial Adipose Tissue Sample

During cardiothoracic surgery, a small sample of epicardial adipose tissue (approximately 15 mg) is collected, which will be immediately snap-frozen in liquid nitrogen and stored at − 80 °C.

### Tissue Analysis

In order to study secondary objectives, serum samples, epicardial adipose, and atrial tissue samples are used to investigate the influence of GGA on HSPs levels, by assessing levels of HSPA1A, HSPB1, and phosphorylated HSF1, as well as the degree of structural remodeling using (confocal) microscopic imaging.

### Safety Laboratory Tests

In order to monitor the previously described liver enzymes’ elevation, from the first and last blood samples, the alkaline phosphatase, gamma-GT, ALT (alanine transaminase, SGPT), AST (aspartate transaminase, SGOT), and bilirubin total and fractionated are determined.

### Primary Endpoints

Holter data are used to identify the incidence of PoAF longer than 10 s.

### Secondary Endpoints

All collected tissue samples will be analyzed to assess levels of HSPB1, HSPA1A, and phosphorylated HSF1, and proteostasis markers, as well as the degree of structural remodeling using (confocal) microscopic imaging.

## Data Management

### Data Collection

Clinical data is collected and securely stored on an encrypted drive. Daily data transmitted via remote monitoring is downloaded from a password-protected cloud database. This data is preserved for 25 years after study completion. The data is used solely for the GENIALITY study, as detailed in the informed consent form.

### Confidentiality

Collected data is secured against unauthorized access. A unique study code is assigned to each participant. The study is conducted in compliance with the guidelines of Good Clinical Practice, in full conformance with the Declaration of Helsinki, and the law on Medical Research involving Human Subjects.

## Statistical Analyses

Regarding clinical characteristics from the study population, such as indication for cardiothoracic surgery, age, sex, BMI, hypertension, diabetes mellitus II, and statin intake, descriptive statistics of continuous variables will be presented as means with standard deviations (SD) or medians with interquartile ranges (IQR), depending on the distribution of the data. Categorical data will be presented as proportions and numbers.

The statistical efficacy analysis is conducted according to the intention-to-treat principle with a chi-square test for proportions of PoAF incidence until D3, between the treatment arms. All randomized patients are included in the primary endpoint analysis. We use the Cox-Mantel test in order to calculate survival rates (PoAF as outcome) until hospital discharge between treatment arms.

HSP levels are continuous measures assessed on adipose tissue and atrial tissue. Adipose and atrial tissue are collected once during surgery. Here, the comparison between control and intervention is cross-sectional, and individual measurements are independent. They will be analyzed using linear regression to compare study arms while adjusting for stratification factors (sex, age, and cardiac surgery indication). HSP levels are also continuous measures assessed on blood samples which are collected at three time points: baseline appointment, at day of surgery, and at day 3 after surgery (see Fig. 1). Therefore, we use statistics suited to longitudinal data, as these are thus dependent observations (correlated within patients). A linear mixed model with at least random intercepts, and, if required, random slopes for time and/or treatment, will be used to compare study arms while adjusting for stratification factors. To preserve the overall type I error rate over all secondary endpoints, Bonferroni correction will be used.

Values of *p* < 0.05 are considered statistically significant. SPSS version 20 (IBM Analytics), R Statistical Software (R Studio, Inc., Boston, MA, USA; version 1.0.153), and GraphPad Prism version 8.0 (GraphPad Software Inc., CA) are used for all statistical evaluations.

### Randomisation and Blinding

For randomization, we consider a 1:1 allocation ratio. Stratified random sampling will take into consideration the following: age (18–60 years or 60–80 years old), sex (male and female), and indication for cardiothoracic surgery (valve disease, CABG, and concomitant valve disease + CABG). Therefore, 12 strata will be considered. A random block size of 4 will be considered.

Stabilization of atrial cardiomyocyte proteostasis appears to be an upcoming and promising therapeutic strategy to avert the AF onset. The GENIALITY trial is the first clinical study to investigate whether HSP-boosting therapy can clinically prevent the incidence of AF in patients undergoing cardiothoracic surgery. The study outcomes will also contribute to a better understanding of the HSP pathway under therapeutic intervention, fostering future investigations with GGA derivatives.

## Conclusion

Stabilization of atrial cardiomyocyte proteostasis appears to be an upcoming and promising therapeutic strategy to avert the AF onset. The GENIALITY trial is the first clinical study to investigate whether HSP- boosting therapy can clinically prevent the incidence of AF in patients undergoing cardiothoracic surgery. The study outcomes will also contribute to a better understanding of the HSP pathway under therapeutic intervention, fostering future investigations with GGA derivatives.

## Data Availability

The data underlying this article will be shared on reasonable request to the corresponding author.
